# Response to the Letter to the Editor by Finsterer and Zarrouk-Mahjoub in Clin Nephrol Case Stud. 2018; 6: 1.

**DOI:** 10.5414/CNCS109366R

**Published:** 2018-01-16

**Authors:** Kenneth Lim, David Steele, Andrew Fenves, Ravi Thadhani, Eliot Heher, Amel Karaa

**Affiliations:** 1 Division of Nephrology and; 2 Department of Genetics and Pediatrics, Massachusetts General Hospital, Harvard Medical School, Boston, MA, USA

**Keywords:** focal segmental glomerulosclerosis, mitochondrial disease

## Abstract

Not available.

## Response to the Letter by Finsterer and Dr. Zarrouk-Mahjoub

We thank Dr. Finsterer and Dr. Zarrouk-Mahjoub for their interest in our article. 

While the m.3243 A>G point mutation is the most commonly described mutation to date in mitochondrial disorders (MDs) that have been linked to renal disease, an increasing number of other mitochondrial mutations have been associated with kidney disease, as outlined in Table 1 of our article [1]. 

Referencing the heteroplasmy statement by Dr. Finsterer et al., the heteroplasmy level in lymphocytes does not reflect any other tissue heteroplasmy level and as such is not predictive of disease severity [2]. It is accepted that in cases of multisystemic MDs, the heteroplasmy level in blood would be lower than in other affected tissues given the negative selection associated with high blood cell turnover. As such, we believe that the argument, that the heteroplasmy level of 30% would not assign pathogenicity, is not well founded. These levels are also above the quoted 15 – 18% heteroplasmy at which pathogenicity is in general assumed [3, 4]. 

We also disagree that the variant did not segregate with the phenotype. We have not done familial segregation analysis in this case since siblings and father have not volunteered to come to our clinic. The mother was tested and was found to carry the same variant at 10% heteroplasmy in leukocytes with no specific MDs symptoms. The variant in the MT-TW tRNA found in this patient does in fact strongly correlate with his phenotype, and his symptoms are very consistent with other tRNA mitochondrial DNA mutations. The constellation of early-onset hearing loss, diabetes, and brain atrophy with basal ganglia calcifications is highly suggestive of a tRNA disorder and is consistent with other case reports of patient and mother carrying the same variant m.5538 G>A. In fact, in the case report with this variant [5] that Finsterer et al. refer to, muscle biopsy and functional studies were consistent with an MD. 

Moreover, the absence on renal histopathology of hematuria and glomerular basement membrane disruption characteristic of Alport’s syndrome argued against a diagnosis of Alport’s. While we agree that further functional studies would be helpful in determining the exact pathogenicity of this variant, overall, given the information we had and the lack of other concerning variants that could explain the phenotype, we strongly believe that this tRNA variant is the etiology for our patient’s presentation. With reference to “nephrolithiasis, and renal cysts being the most frequent renal manifestations in MDs”, aside from the 3 case reports listed in Finsterer and Scorza’s recent publication [6], we do not see evidence of this being as common. In fact, even in his own publication, in Table 1, these are present in only 1 and 3 patients, respectively. For a disease that has a prevalence of 1 : 5,000 in adults, these manifestations seem to be very rare. Our patient did not have any other real involvement aside from the FSGS. 

Serum lactate levels can be elevated or normal in patients with suspected MD, as in our patient [7]. The absence of an elevated lactate on stress testing does not rule out an MD, and therefore the patient did not undergo this test. As noted in our report, a limitation of our case presentation is that we were unable to obtain tissue for functional analysis, and a muscle biopsy was declined. 

Dr. Finsterer et al. raised the question of cardiovascular risk factors in the setting of our patient’s history of prior stroke. As already described in our case report, the patient did not use tobacco, and he did not have a history of atrial fibrillation, hyperlipidemia, or heart failure. The family history was illustrated in Figure 1 of the article and was significant for only hypercholesterolemia in his father [1]. Dr. Finsterer et al. raised the question of long-term post-transplant follow-up. As already noted in the article, our patient has had an uncomplicated post-transplant course since living related kidney transplantation from his father. 

In summary, the authors believe that in the case presented, the MT-TW tRNA change at position m.5538 G>A is most consistent with the described pre sentation. As clearly stated in our report, we provide genotype-phenotype evidence of an FSGS-associated MD at this position, and we agree that further studies are needed to establish causality. 

## Conflict of interest 

All authors declare no conflicts of interests. 

## Funding 

None. 
